# Determination of Aflatoxins in Milk by PS-MWCNT/OH Composite Nanofibers Solid-Phase Extraction Coupled with HPLC-FLD

**DOI:** 10.3390/molecules28166103

**Published:** 2023-08-17

**Authors:** Lanlan Wei, Yanan Chen, Dongliang Shao, Jingjun Li

**Affiliations:** 1 College of Food Engineering, Anhui Science and Technology University, Chuzhou 233100, China; 18356130656@163.com (L.W.); ynchen16@163.com (Y.C.); 2 Anhui Guoke Testing Technology Co., Ltd., Hefei 230000, China; 17855348740@163.com

**Keywords:** aflatoxins, packed-nanofiber solid-phase extraction (PFSPE), sample preparation, polystyrene polymeric multi-walled carbon nanotube (PS-MWCNT/OH), milk

## Abstract

In this work, a sensitive analytical method based on packed-nanofiber solid-phase extraction (PFSPE), after derivatization with trichloroacetic acid and high-performance liquid chromatography with a fluorescence detector (HPLC-FLD), has been established for the determination of aflatoxins (AFs) in milk. Polystyrene polymeric multi-walled carbon nanotube (PS-MWCNT/OH) composite nanofibers were fabricated by electrospinning and used to prepare homemade extraction columns. The extraction efficiency of the HPLC-FLD analysis method was sufficiently investigated and validated. After the implementation of optimal conditions, all of the analytes were separated efficiently and the components of the milk matrix did not disturb the determination. The obtained linear ranges of the calibration curves were 0.2–20 ng/mL for AFTB1 and AFTG2, 0.1–10 ng/mL for AFTB2, and 0.4–40 ng/mL for AFTG1. The recoveries ranged between 80.22% and 96.21%. The relative standard deviations (RSDs) for the intra-day and inter-day results ranged from 2.81–6.43% to 3.42–7.75%, respectively. Generally, 11 mg of sorbent and 200 μL of elution solvent were used to directly extract all of the AFs from the milk matrix. Reported herein is the first utilization of PS-MWCNT/OH-PFSPE HPLC-FLD to simultaneously analyze the occurrence of aflatoxins in milk.

## 1. Introduction

Aflatoxins (AFs) are secondary metabolites produced mainly by the mold fungi of the genus *Aspergillus* (A.), such as *A. flavus*, *A. parasiticus*, and *A. nomius* [1]. Many animal species are susceptible to carcinogenic, mutagenic, teratogenic, and immunosuppressive effects by these mycotoxins. Yet, aflatoxins cannot be completely dislodged by current agricultural practices and food processing owing to its wide availability in commonly-consumed food commodities such as cereals, peanuts, and dairy products [2,3]. More seriously, aflatoxins lead to health problems upon accumulating within the human body through the food chain [4,5,6]. So far, aflatoxin B1 (AFB1), aflatoxin B2 (AFB2), aflatoxin G1 (AFG1), and aflatoxin G2 (AFG2) occupied the main position, of which about 20 kinds of AFs have been identified.

The reason why a sensitivity analysis determination is needed is that there are trace amounts of mycotoxin biomarkers in physiological samples. Additionally, sample preparation is critical for effective analyte detection [7]. The literature has reported many kinds of methods to detect AFs in food and animal feed; these involve capillary electrophoresis [8], thin-layer chromatography [9], high-performance liquid chromatography (HPLC) [10], and enzyme-linked immunosorbent assay [11]. At present, high-performance liquid chromatography with a fluorescence detector (HPLC-FLD) has been widely attractive for AF detection in food due to its simplicity, feasibility, and accuracy.

In order to purify the matrix, extract, and preconcentrate target substances, it is key to find an appropriate sample pretreatment method to solve the problems of complex sample components and low analyte concentration. Various pretreatment methods have been employed to extract AFs from food matrices, such as liquid–liquid extraction (LLE) [12], dispersive liquid–liquid microextraction (DLLME) [13], molecular imprinting (MIP) [14], magnetic solid-phase extraction (MSPE) [15], and immunoaffinity column (IAC) purification [16]. Solid-phase extraction (SPE) is a universally-known method that is effective in preconcentrating and purifying the analytes from environmental, biomedical, and food matrices in controlled laboratory settings through solid–liquid extraction. Silicon, carbon, polymer materials C18 [17], Oasis hydrophilic-lipophilic-balanced (HLB) functionalized through group modification [18], and graphite carbon black (GCB) [19] are all traditional extraction materials.

Among the advantages of packed-fiber solid-phase extraction (PFSPE) is the reduction of the traditional process of sample pretreatment through integrating purification, concentration, and desorption into one step without other processes like film filtering, evaporation by heat, and drying by nitrogen stream. As an extraction medium, electrospinning nanofibers have a small adsorption bed mass and thus reduce the amount of organic solvents required during extraction. Yet, the main reasons why nanofibers improve the extraction efficiency of trace analysis are due to their unique physical and chemical properties, such as large surface area, high porosity, and flexibility of chemical/physical modification [20]. Therefore, electrospinning nanofibers are treated as appropriate potential adsorbing materials applied to SPE-based techniques [21,22]. It is important that the carbon nanotubes (CNTs) are applied to treat solid-phase extraction (SPE) and solid phase microextraction (SPME) sorbents because the inorganic and organic compounds can be extracted upon the non-polar substances (mainly extracted with non-functionalized CNTs) and polar molecules (extracted with functionalized CNTs), respectively. The innermost tubular layer of multi-walled carbon nanotube (MWCNT) diameters ranged from 2 to 10 nm, and meanwhile the additional thickness of every extra layer was about 0.7 nm. Above all, MWCNTs can be either semiconducting or metallic with high electrical conductivities (~105 Scm^−1^). Fang et al. [23] used multi-walled CNTs (MWCNTs) with an average diameter between 60 and 100 nm and 5–15 μm of length to extract ten SAs (i.e., sulfadiazine (SDZ), sulfamerazine (SMR), sulfadimidin (SDD), sulfathiazole (STZ), sulfamoxol, sulfametizole, sulfamethoxypyridazine (SMP), sulfachloropyridazine, sulfadoxine (SDX), and sulfisoxazole (SSZ)) from eggs and pig tissues by on-line SPE combined with HPLC-UV. Polystyrene (PS) was chosen as the matrix material due to its well-known properties, including ease of processing, good solubility in a broad range of solvents, and its clarity that enables the observation of MWCNT dispersion at the micron scale. Moreover, not only were there no chemical pretreatments, purifications, and modifications needed to prepare MWNCTs, but these can also be dispersed by ordinary ultrasonication treatment.

In this study, polystyrene polymeric multi-walled carbon nanotube (PS-MWCNT/OH) functional nanofibers used as absorbent materials were prepared and characterized. A packed PS-MWCNT/OH-electrospinning-nanofiber SPE, coupled with high-performance liquid chromatography with a fluorescence detector (HPLC-FLD), were used to detect four aflatoxins, namely AFB1, AFB2, AFG1, and AFG2 in pure milk samples. In addition, other important parameters that affect extraction efficiency, including eluent volume, amount of extracted materials, and stability of the PS-MWCNT/OH nanofibers, were investigated and optimized. Finally, the four aflatoxins in the milk matrix were detected by this established method.

## 2. Results and Discussion

### 2.1. Characterization of Nanofibers

**Figure 1:**
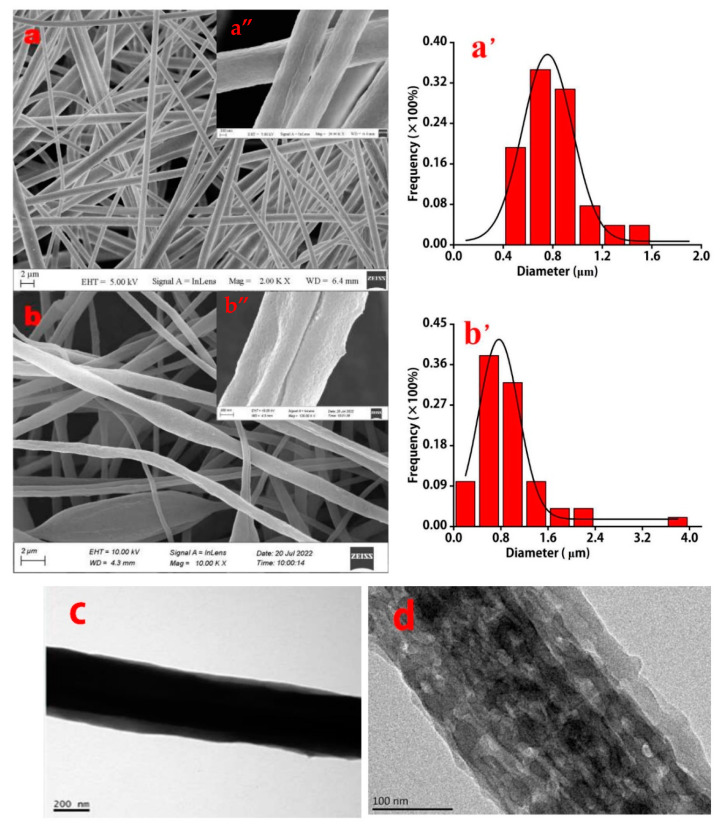
Characterization of electrospinning nanofibers by SEM (**a**,**a″**,**b**,**b″**), in addition the ruler length of the SEM (**a″**,**b″**) is 200 nm, TEM (**c,d**), and the size distributions of nanofibers (**a′**,**b′**). (**a**,**c**) 10% (*w*/*v*) PS single nanofibers; (**b**,**d**) 10% PS-10% HMWCNTs (*w*/*v*) composite nanofibers.

Compared with the SEM images of PS nanofibers, those of PS-MWCNT/OH nanofibers showed no significant morphological change (Figure 1a,b). In addition, the PS-MWCNT/OH nanofibers exhibited a rougher surface than PS nanofibers. As shown in Figure 1a′,b′, the PS and PS-MWCNT/OH nanofibers’ mean diameters were 0.8 μm and 0.91 μm. In addition, the structures of the nanofibers looked like networks and were uniform and dense. The PS-MWCNT/OH nanofibers had no obvious beads, and there were traces of particle attachments on the fiber surface, indicating the binding of polystyrene and hydroxylated multi-walled carbon nanotubes.

Compared with PS nanofibers, the spots intersected with light and dark on the PS-MWCNT/OH composite nanofibers, as shown by the TEM images of Figure 1a,b, indicated that there were large numbers of nanopores. Moreover, the surface area of the composite nanofibers can be obviously increased by these numerous special porous structures.

### 2.2. Fourier-Transform Infrared Spectroscopy Analysis

**Figure 2:**
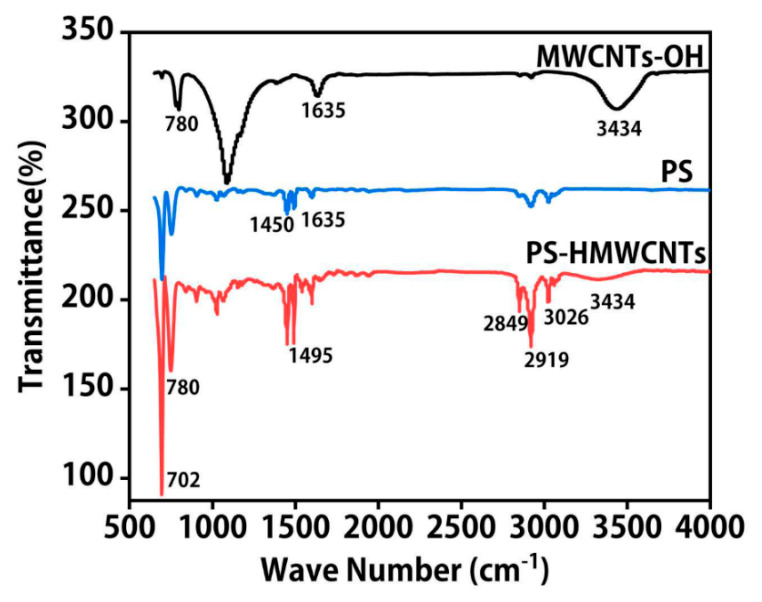
FTIR spectra of PS, MWNCTs-OH and PS-MWNCTs-OH composite nanofibers.

The Fourier-transform infrared (FTIR) spectra of the PS and PS-MWNCTs-OH nanofibers are shown in Figure 2. The peaks at 694 cm^−1^ and 752 cm^−1^ were assigned to the C–H vibrational absorption peaks of the PS benzene ring. However, the peaks at 1026 cm^−1^ and 1450 cm^−1^ were attributed to the C–H bending and C–H deformation vibrations, respectively. Both peaks at 1492 cm^−1^ and 1600 cm^−1^ were due to the C=C double-bond stretching vibration peaks. The absorption peaks at 2849 cm^−1^, 2918 cm^−1^, and 3024 cm^−1^ were caused by the C–H stretching vibrations. At 1220 cm^−1^, there was a strong absorption peak, which was caused by the C–C stretching vibration of the main structure of the carbon nanotube. The strong absorption peak at 1635 cm^−1^ was assigned to the C=C stretching vibration of the multi-walled carbon nanotube. The characteristic peak of the hydrophilic group appeared in the infrared spectrum of MWNCTs-OH, and the broad and strong peak at 3434 cm^−1^ was the O–H stretching vibration peak of the hydroxyl group, which fully demonstrated the successful hydroxylation modification of carbon nanotubes. There were no new absorption peaks on the infrared spectrum of PS-MWNCTs-OH, indicating that no new chemical bonds were formed.

### 2.3. X-ray Diffraction Analysis (XRD)

**Figure 3:**
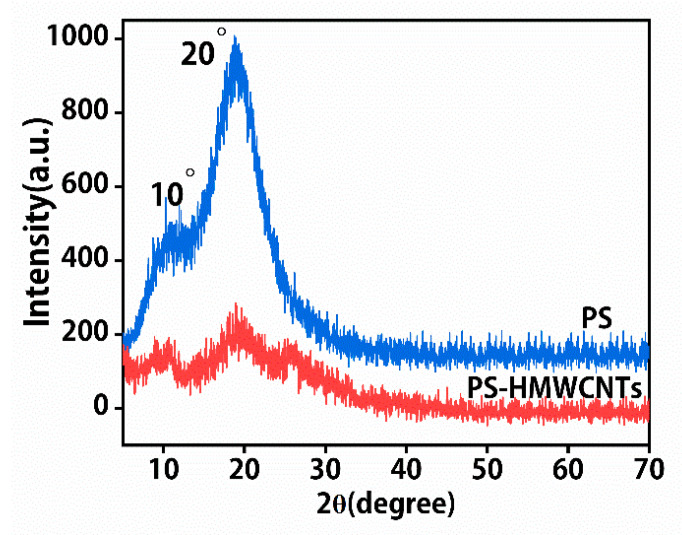
XRD patterns of PS nanofibers and PS-MWCNTs-OH composite nanofibers.

Figure 3 demonstrates the X-ray diffraction analysis of PS nanofibers and PS-HMWCNT composite nanofibers. As shown in Figure 3, the PS nanofibers’ characteristic diffraction peaks were 2θ = 11° and 18°, respectively. The XRD characteristic diffraction peaks of PS-HMWCNT nanofibers were similar to the PS nanofibers, at 2θ = 10° and 20°. In addition, the PS-HMWCNT composite nanofibers’ overall crystallinity was lower than that of PS because the peak intensities of the PS-HMWCNT composite nanofibers at 2θ = 10° and 20° were lower. The above results indicate that pure MWNCTs-OH and PS particles were thoroughly mixed and that strong molecular interactions were generated between them.

### 2.4. Optimization of PS-HMWCNT Nanofibers Packed-Fiber Solid-Phase Extraction

The amount of adsorption material is an important factor affecting the elution rate of aflatoxin. Herein, we investigated the effect of 8–14 mg of PS-MWCNTs-OH nanofibers on the elution rate of aflatoxin. When the amount of filler increased from 8 mg to 11 mg, the elution rate of aflatoxin gradually increased. Subsequently, a further increase in the amount of adsorption material resulted in a decreasing in the elution rate of the target substance. In fact, minimal adsorbent material can lead to incomplete adsorption of the target substance, while abundant use of the adsorbent can hinder elution as more target substances remain on the adsorbent material, thereby reducing the extraction efficiency.

**Figure 4:**
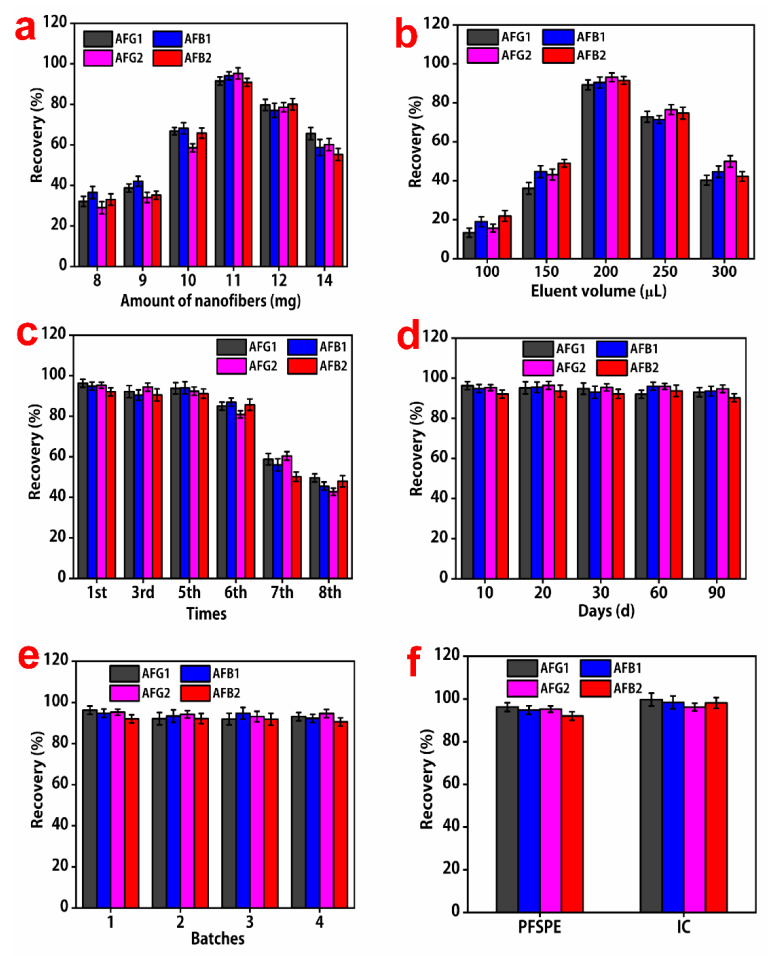
(**a**–**e**) Effects of the amounts of nanofibers, eluent volume, different days, extraction repeats, and different batches on recovery percentage. (**f**) The comparison between PS-MWCNTs-OH nanofibers and immunoaffinity column (IC) on the extraction efficiency at a concentration of 50 ng/mL for the four aflatoxins (AFs).

The good stability of nanofibers was a crucial factor for the elution rate of aflatoxin. To investigate the effect of nanofibers from the same batch of materials on different days, the elution rates of aflatoxin were investigated under the same experimental conditions after 10, 20, 30, 60, and 90 days. As shown in Figure 4, the elution rates of aflatoxin were almost the same for the nanofibers prepared from the same batch at different times. The experimental results showed that the nanofibers had good stability.

Upon repeating the extraction six times using the same PS-MWCNTs-OH nanofiber solid-phase extraction column, there were no significant changes in the elution rates of the aflatoxins. After the seventh repeated extraction, the elution rate obviously decreased. This indicates that the material has good repeatability and stability.

Four batches of PS-MWCNTs-OH nanofibers prepared at different times were used to investigate the effect of different batches of nanofiber materials on the elution rates of the four aflatoxins. Figure 4 depicts that, through the adsorption of nanofibers prepared at different times, the elution rates of the target substance were almost the same. The results illustrated that the quality of PS-HMWCNT nanofibers prepared in different batches maintained good consistency and stability.

Methanol was selected as an eluent to perform optimization experiments with different volumes, including 100 μL, 150 μL, 200 μL, 250 μL, and 300 μL. Upon increasing the volume of methanol from 100 μL to 200 μL, the elution rate of aflatoxin gradually increased. However, the elution rate of aflatoxin slowly decreased upon increasing the volume of methanol to 300 μL (Figure 4b). Therefore, 200 μL was selected as the optimal volume of methanol.

The extraction efficiency of the PS-MWCNTs-OH nanofiber solid-phase extraction column was almost identical to that of the commercial immunoaffinity column (IC), and met the extraction requirements of actual samples. However, using the immunoaffinity column to extract aflatoxin from milk required multiple rinses with water, drying the solid-phase extraction purification column, and then eluting to volume. Accordingly, the IC method was cumbersome, expensive, and lengthy, which was unfavorable for the detection of a large number of samples.

### 2.5. Method Validation

In order to calibrate and verify the developed method, one kind of milk sample with free aflatoxins, as reported before, was detected by the National Standard Method [24] and then treated as a blank sample. Repeatability, linearity, limit of detection (LOD), and LOQ were obtained by the optimized conditions. As shown in Table 1, good linearity was found in the range of 0.5–20.0 ng mL^−1^ for AFTB, 0.5–20.0 ng mL^−1^ for AFTG2, 0.2–10 ng mL^−1^ for AFTB2, and 0.5–40 ng mL^−1^ for AFTG1. The LODs and LOQs of AFTB1, AFTG2, AFTB2, AFTG1 ranged from 0.07–0.16 ng mL^−1^ to 0.23–0.53 ng mL^−1^, respectively. The low, medium, and high concentrations of 0.05, 0.5, and 5 ng/mL were added into the blank milk as the spiked samples detected by the new established method and calculated to get intra-day and inter-day precisions. Hence, the intra-day RSD was 2.81–6.43% and the inter-day RSD was 3.42–7.75%. The recoveries of the four aflatoxins in different concentrations ranged from 80.22% to 96.21%. Generally, the above results indicated that this method is appropriate for detecting aflatoxins in milk samples.

### 2.6. Comparison of Methods

As shown in Table 2, though the proposed PFSPE coupled with the HPLC-FLD method for AFTB1, AFTG2, AFTB2, and AFTG1 quantification was compared with the other developed methods from the literature, the advantages involved less usage of organic solvent, time, and cost. It was clear that the new method established for this paper was sensitive and convenient enough to detect AFTB1, AFTG2, AFTB2, and AFTG1 in milk samples. The reason why the new method was different from the other SPE methods was that extraction, purification, enrichment, and elution were integrated to achieve the effect of enrichment instead of nitrogen evaporation and redissolution. From the very beginning, PS-MWCNTs-OH electrospinning nanofibers were treated as the adsorbent materials. As a result, the proposed PFSPE method is suitable to be applied to sample pretreatment due to being less time consuming, easier to conduct, and more environmentally friendly. In addition, a noted limitation was that it is usually required to manually fill nanofibers into the extraction column. In order to make up for this deficiency, the development of commercial standardization is required.

### 2.7. Application to Real Samples

**Figure 5:**
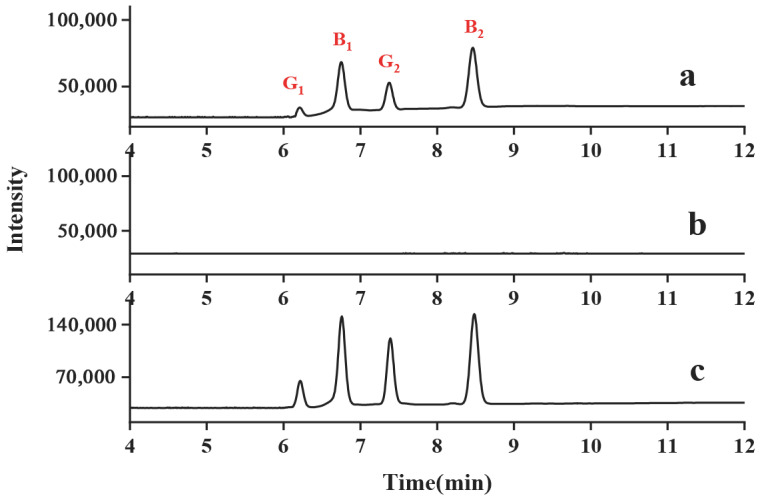
Typical HPLC chromatograms of (**a**) blank milk consisting of 10 ng mL^−1^ of aflatoxins with pretreatment using the PS-MWCNTs-OH nanofibers, (**b**) milk with pretreatment using the PS-MWCNTs-OH assembled nanofibers, and (**c**) standard solution consisting of 10 ng mL^−1^ of aflatoxins.

In order to evaluate the applicability of the proposed method in real samples, the method was applied to determine the blank and spiked milk samples. Figure 5 demonstrated the four aflatoxin (AFTB1, AFTG2, AFTB2, and AFTG1) standard HPLC-PFSPE-FLD chromatograms with a concentration of 10 ng mL^−1^ spiked in blank milk. The main reason why the chromatogram peaks of four aflatoxins was sharp and narrow with baseline resolutions is due to the good adsorption of the PS-MWCNTs-OH nanofibers adsorbent for the target compounds. From the chromatogram displayed, not only can the target compounds be extracted by the PS-MWCNTs-OH nanofibers, the interference can also be removed effectively and matrix interference reduced. The results indicated that the established method can be applied to quantitative analyses of AFTB1, AFTG2, AFTB2, and AFTG1 in milk samples.

## 3. Materials and Methods

### 3.1. Materials and Reagents

Milk (Fengyang Supermarket) and polystyrene (PS, retained molecular weight: 18,500 Da) were purchased from the Aladdin company (Shanghai, China, www.aladdin-e.com). Multi-walled carbon nanotubes (MWCNTs-OH) were obtained from Suzhou Dongqi Biotechnology Co., Ltd. (Suzhou, China). Methanol (chromatographically pure) was purchased from Shanghai Aladdin Biochemical Technology Co., Ltd. (Shanghai, China). The solvents acetonitrile (chromatographic grade), trifluoroacetic acid (chromatographic grade), and Formic acid (85%, analytical purity) were obtained from Shanghai McLean Biochemical Technology Co., Ltd. (Shanghai, China).

Aflatoxin standard products, namely aflatoxin B1 (AFTB1), aflatoxin B2 (AFTB2), aflatoxin G1 (AFTG1), and aflatoxin G2 (AFTG2), where obtained with purity ≥98% from Shanghai McLean Biochemical Technology Co., Ltd. (Shanghai, China).

### 3.2. Experimental Instruments

Japan Shimadzu LC-20A high-performance liquid chromatograph was equipped with a LC-20AT four-element pump, CT0-20A column temperature box, CBM-20A controller, SIL-20A automatic sampler, and a fluorescence detector (Japan Shimadzu Company, Kyoto, Japan). Among the other instruments utilized during this study are the KQ3200E ultrasonic cleaner (Kunshan Ultrasonic Instrument Co., Ltd., Shanghai, China), vortex-M vortex mixer (Shanghai Huxian Industrial Co., Ltd., Shanghai, China), DHG-9030A electric thermostatic blast drying oven (Shanghai Sanfa Scientific Instrument Co., Ltd., Shanghai, China), Mettler XS105DU electronic balance (Mettler Toledo Instruments Shanghai Co., Ltd., Shanghai, China), FD.JH.JS 1000 mL ultrafiltration device, AP-01P vacuum pump (Tianjin Otsaines Instrument Co., Ltd., Tianjin, China), TS-110X50 reciprocating water bath constant temperature shaker (Shanghai Tiancheng Experimental Instrument Manufacturing Co., Ltd., Shanghai, China), TGL-16 desktop high-speed freezing centrifuge (Hunan Xiangyi Laboratory Instrument Development Co., Ltd., Changsha, China), Nicolet iS 10 Fourier-transform infrared spectrometer (Thermo Fisher, Waltham, MA, USA), XD-3X ray diffractometer (Beijing General Analysis Instrument Co., Ltd., Beijing, China), DSC-3 differential scanning calorimeter (METTLER TOLEDO, Zurich, Switzerland), Supra55 field emission scanning electron microscope (Zeiss, Oberkochen, Germany), and JEM-2100F high-resolution transmission electron microscope (JEOL Company, Kyoto, Japan).

### 3.3. Preparation of Standard Solution

Accurately, 1.0 mg (±0.1 mg) was weighed for each of AFTB1, AFTB2, AFTG1, and AFTG2 into a 10 mL brown volumetric flask. The AFs were dissolved with methanol to prepare standard stock solutions with concentrations of 100 μg mL^−1^. The AFTG1 sample was diluted with distilled water to concentrations of 0.4 ng mL^−1^, 2 ng mL^−1^, 10 ng mL^−1^, 25 ng mL^−1^, and 40 ng mL^−1^. The concentrations of AFTB1 and AFTG2 after dilution were 0.2 ng mL^−1^, 1 ng mL^−1^, 2.5 ng mL^−1^, 10 ng mL^−1^, and 20 ng mL^−1^, while the concentrations of AFTB2 were 0.1 ng mL^−1^, 0.5 ng mL^−1^, 2 ng mL^−1^, 5 ng mL^−1^, and 10 ng mL^−1^.

The intra-day and inter-day precision and accuracy were evaluated for pure milk samples spiked with AFs (AFTB1, AFTB2, AFTG1, and AFTG2; *n* = 3) at concentrations of 0.5, 2, and 5 ng mL^−1^ according to the above pretreatment method. The inter-day precision was evaluated on six successive days. The precision of the method was reported as the RSD of repeatability at each analyte concentration.

### 3.4. Fabrication of Electrospinning Polystyrene Polymeric Multi-Walled Carbon Nanotube (PS-MWCNT/OH) Composite Nanofibers

Firstly, PS (10%, *w*/*v*) was dissolved in the mixed solvent DMF and THF (4:6, *v*/*v*) by magnetic stirring to prepare the electrospinning solution. Secondly, after the PS completely dissolved, multi-walled carbon nanotubes (MWCNT-OH) (10%, *w*/*v*) were added to the polymer solution and stirred for more than 10 h at room temperature before electrospinning.

Then, a glass syringe was filled with the solution; the syringe was installed with a steel needle with the tip diameter of 0.5 mm and the tip was full of the precursor solution flat with a rate of 2.0 mL h^−1^. A piece of aluminum foil paper was used as the collector, and the polymer solution was electrospun at a positive voltage of 24 kV (+24 kV was applied to the syringe needle and 0 V was applied to the collector). The distance from the needle tip to the collector was 20 cm.

### 3.5. Sample Pretreatment and Extraction

The milk sample was purchased from a local supermarket. Only 5.0 g (±0.01 g) of the sample was weighed into a 50 mL plastic centrifuge tube. Then, 15 mL of 70% acetonitrile and 5 mL of 0.1% formic acid were added and the solution was placed in an ultrasonic cleaner for 20 min. Subsequently, the sample was frozen and centrifuged at 9000 r min^−1^ for 10 min, followed by the taking of the supernatant.

In order to extract targets, firstly 11.0 mg of nanofibers were added into the SPE columns arrived at the tip ends treated as the adsorbing materials; secondly, in order to activate the PFSPE columns successfully, 200 μL methanol and 200 μL water were added in proper order. Finally, the 500 μL target extraction was isolated from the nanofiber solid-phase extraction column and eluted with 200 μL pure methanol. Then, 200 μL of elution was added into a 10 mL centrifuge tube and dried in an oven at 50 °C, then 200 μL trifluoroacetic acid was added and swirled for 1.0 min and derivatized in a water bath shaker at 40 °C for 30 min. After the derivatization was completed, the filtrate was collected and placed in an injection bottle for injection with HPLC [18]. During the whole extraction process, the samples were carefully controlled to flow through the PFSPE extraction column at a slow and uniform flow rate. In particular, a rapid flow rate yields an incomplete extraction, while a very slow flow rate blocks the extraction, resulting in the sample to be measured remaining in the PFSPE extraction column. The extraction recovery was calculated using the following equation:

q = (A_1_/2.5/A_0_) × 100%, (1)

where A_0_ and A_1_ are the peak areas of 10 μL aflatoxin standard solutions and eluent solutions, respectively.

### 3.6. Chromatographic Conditions

The Shimadzu series high-performance liquid chromatography (www.shimadzu.co.jp, Shimadzu, Kyoto, Japan) consisted of two parts, involving an analytical column (Wondersil C18, 250 mm × 4.6 mm, 5 μm) and a fluorescence detector. Mobile phase: 60% methanol solution (*v*/*v*) (60:40), flow rate: 1.0 mL min^−1^; the wavelengths of excitation and emission were set at 360 nm and 440 nm, respectively. The retention time, structure, formula and molecular weight for each analyte are listed in Table 3.

### 3.7. Characterization

The surface morphologies of the electrospinning fibers were observed using a scanning electron microscope (SEM, Supra55, ZEISS, Germany) at 20 kV. The average diameter of the electrospinning fibers was determined by analyzing 30 single nanofibers using the nano measurer software (Nano Measurer 1.2, China). The molecular structure of the nanofibers’ membranes was confirmed by ATR-IR spectroscopy (FTIR, NicoletiS10, Thermo Fisher, USA), and carried out in the range of 4000–500 cm^−1^. Transmission electron microscopy (TEM) images were taken with a JEM-2010 microscope. The X-ray diffraction (XRD) characterized was performed by an X-ray diffractometer (XD-3X, Persee General, Shanghai, China), with the XRD spectra 2θ ranging from 5° to 60°, and the step size was 0.02°.

## 4. Conclusions

In this paper, the PS-MWCNT/OH composite nanofibers as absorbent materials couple with the HPLC-FLD method was developed to simultaneously detect four aflatoxins (AFTB1, AFTG2, AFTB2, and AFTG1) in milk samples. The good linearity, precision, LOD, and LOQ of this method were obtained under the optimized conditions. This analytical method showed high accuracy, with recoveries between 80.22 and 96.21%. The RSDs of intra-day and inter-day ranged from 2.81–6.43% to 3.42–7.75%, respectively. The application area of nanofibers as adsorbent materials can be extended in accessible and efficient extraction devices though the development technique of fabricating selective nanofibers. More importantly, compared with traditional sample pretreatment methods, evaporative drying under nitrogen or membrane treatment were not required in this established sample pretreatment method. Hence, our proposed novel method is timesaving, economical, environmentally friendly, feasible, and easy to generalize.

## Figures and Tables

**Table 1:** Performance parameters of the proposed method upon extracting the four AFs.

Analyte	Linearity Range (ng mL^−1^)	LOD (ng mL^−1^)	LOQ (ng mL^−1^)	Recovery (%) ± RSD (*n* = 3)		
				Added (ng mL^−1^)	Intra-Day	Inter-Day
AFTB1	0.5~20	0.13	0.44	0.5	88.85 ± 4.45	86.63 ± 6.41
				2	90.13 ± 3.86	90.23 ± 4.14
				5	91.34 ± 3.63	91.47 ± 3.42
AFTB2	0.2~10	0.07	0.23	0.25	89.66 ± 6.43	89.82 ± 5.60
				1	90.49 ± 3.51	91.19 ± 3.94
				2.5	91.40 ± 2.99	92.45 ± 4.28
AFTG1	0.5~40	0.16	0.53	1	89.45 ± 4.43	88.46 ± 7.75
				5	90.09 ± 3.08	89.32 ± 3.43
				20	91.01 ± 2.81	90.88 ± 4.04
AFTG2	0.5~20	0.13	0.45	0.5	88.06 ± 4.13	88.92 ± 5.39
				2	89.80 ± 3.61	91.15 ± 3.96
				5	91.06 ± 3.49	91.56 ± 3.77

**Table 2:** Comparison of this technique with methods from the literature for detecting aflatoxins in food.

Extraction Methods	Detection Methods	Derivation Time	Recovery	LOD	Linear Range	Target	Real Sample	References
Extract DNA and PCR	ELISA	/	/	<1 ng mL^−1^	0~1.0 ng mL^−1^	AFTB1	Peanut, wheat flour, milk powder	[25]
DLLME	HPLC-PCD	/	82.0~104.0%	/	0.5~10.0 ng mL^−1^	AFTB1, AFTG2, AFTB2, and AFTG1	Oat, rice, coconut, almond, and birdseed plant-based milk and milk-based products enriched with oats, almonds, and walnuts	[26]
SPE	LC-MS/MS	/	87.4–102.1%	0.05 ng mL^−1^	0.1~20 ng mL^−1^	AFTB1	Milk	[27]
/	LC-MS/MS	/	/	0.05 ng mL^−1^	0.01–2.5 ng mL^−1^	AFTB1, AFTG2, AFTB2, and AFTG1	Infant formula	[28]
PFSPE	HPLC-RF	30 min	86.63~92.45%	0.03~0.08 ng mL^−1^	0.2~40 ng mL^−1^	AFTB1, AFTG2, AFTB2, and AFTG1	Milk	This method

**Table 3:** The parameters for each analyte.

Analyte	Structure	Formula	Molecular Weight (Da)	Retention Time (min)
AFTG1	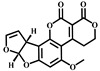	C_17_H_12_O_7_	328.27	6.217
AFTB1	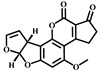	C_17_H_12_O_6_	312.27	6.758
AFTG2	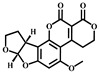	C_17_H_14_O_7_	330.29	7.390
AFTB2	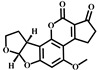	C_17_H_14_O_6_	314.29	8.843

## Data Availability

Data are contained within the article.
